# Supplementary material for: Higher Antarctic ice sheet accumulation and surface melt rates revealed at 2 km resolution
Source: Nat Commun. 2023 Dec 1;14:7949. doi: 10.1038/s41467-023-43584-6 (PMC10692123; doi:10.1038/s41467-023-43584-6)
Supplement: Supplementary file 1 — Supplementary Information [file 41467_2023_43584_MOESM1_ESM.pdf]

Supplementary Information: "Higher Antarctic ice sheet accumulation and surface melt rates revealed at 2 km resolution"

Brice Noël*[1,2], J. Melchior van Wessem[1], Bert Wouters[3], Luke Trusel[4], Stef Lhermitte[5,3], and Michiel R. van den Broeke[1]

[1] *Laboratoire de Climatologie et Topoclimatologie, University of Liège, Liège, Belgium.*
[2] *Institute for Marine and Atmospheric research Utrecht, Utrecht University, Utrecht, Netherlands.*
[3] *Department of Geoscience & Remote Sensing, Delft University of Technology, Delft, Netherlands.*
[4] *Department of Geography, Pennsylvania State University, University Park, PA, USA.*
[5] *Department of Earth & Environmental Sciences, KU Leuven, Leuven, Belgium.*

Corresponding author: Brice Noël (bnoel@uliege.be)

This document includes:

- Supplementary Figures 1-7
- Supplementary Tables 1-3
- Supplementary References

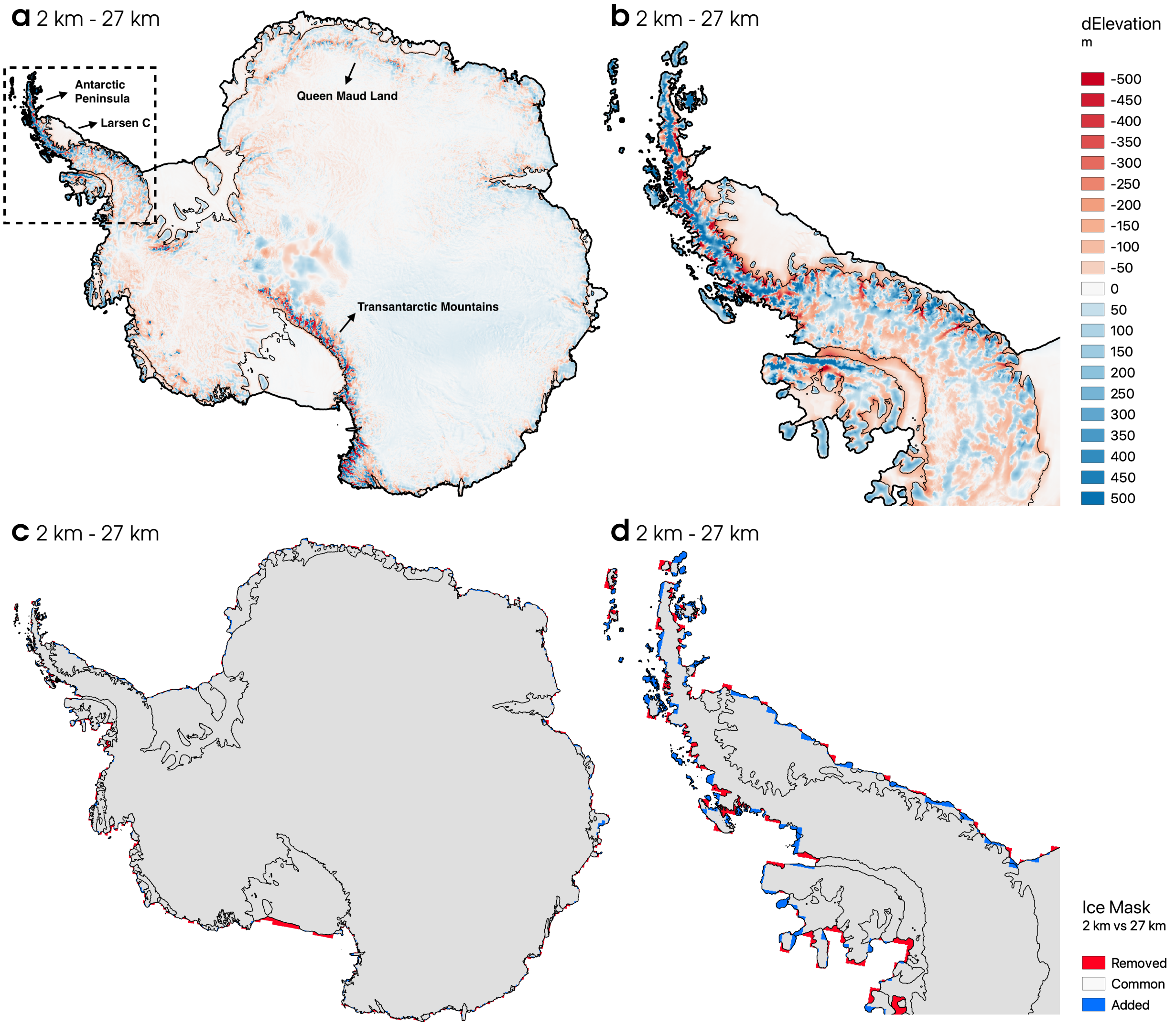

Supplementary Figure 1: Elevation and ice mask difference in a low-resolution climate model. Elevation difference between the high-resolution digital elevation model REMA[1], down-sampled to 2 km, and RACMO2.3p2 at 27 km spatial resolution[2] over **a** the whole of Antarctica, and **b** the Antarctic Peninsula. **c** and **d** Difference in ice mask between the 2 km and 27 km grids. The common ice mask is shown in grey, added and removed ice pixels in the 2 km grid are displayed in blue and red, respectively. Black contour lines in **a-d** represent the ice sheet and ice shelves extent at 2 km.

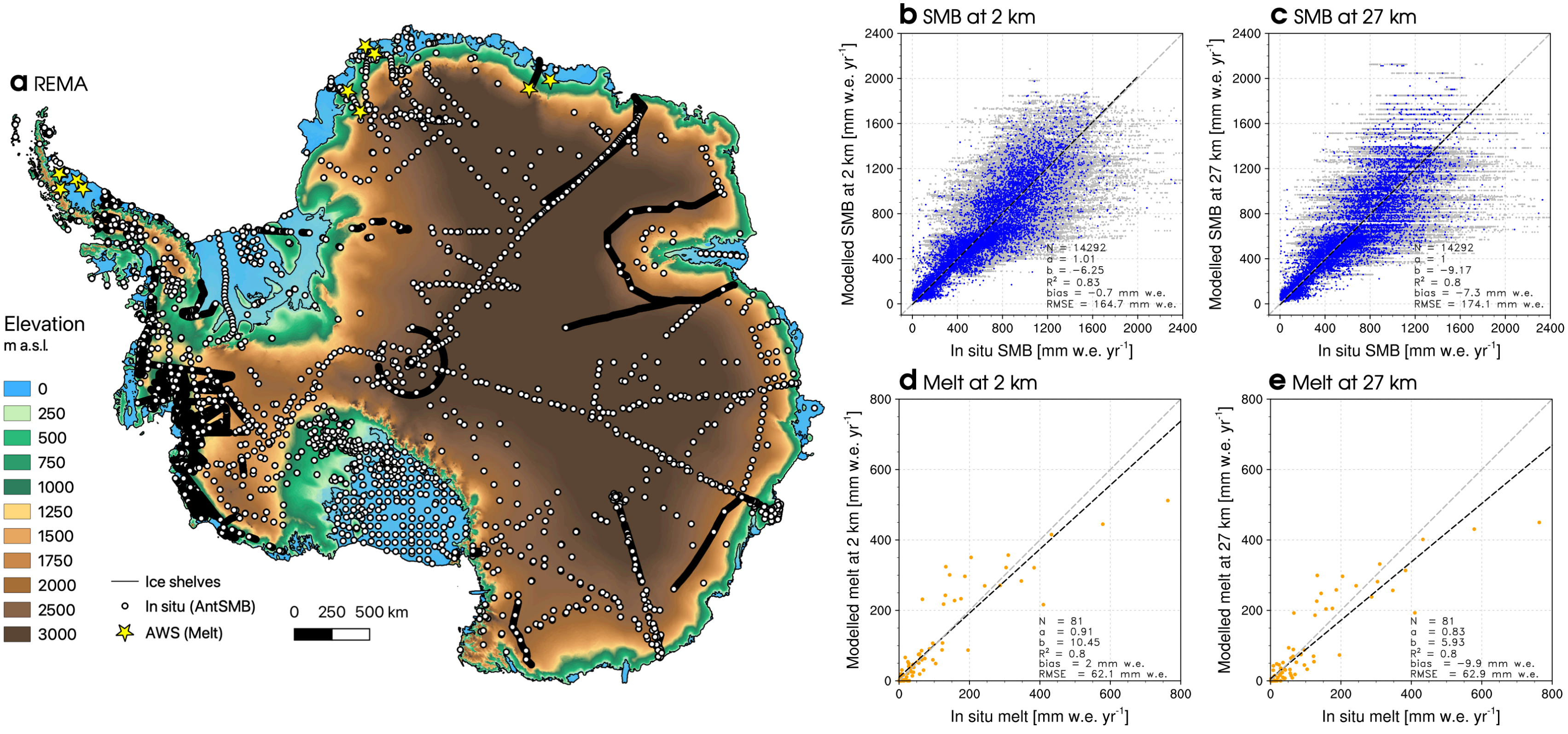

Supplementary Figure 2: Model evaluation of accumulation and melt using in-situ records. **a** Surface topography from the digital elevation model REMA[1] down-sampled to 2 km. White dots and yellow stars locate the in-situ surface mass balance (SMB) and automatic weather stations (AWS) used for model evaluation. In-situ SMB sites (dots) that are situated very close to each other form thick black lines (overlapping dots). Ice shelves are outlined with a cyan contour. Evaluation of modelled SMB **b** at 2 km and **c** 27 km spatial resolution with in-situ measurements from the AntSMB data set[3]. Blue dots in **b-c** account for spatial averaging of in-situ measurements located within a single 2 km grid-cell, while grey dots show the raw data set. Evaluation of modelled melt **d** at 2 km and **e** 27 km spatial resolution with in-situ measurements from ten AWS[4]. Relevant statistics including number of records (N), the slope (a) and intercept (b) of the regression line (y = ax + b), coordination coefficient (R^2), mean model bias and root mean square error (RMSE) are listed.

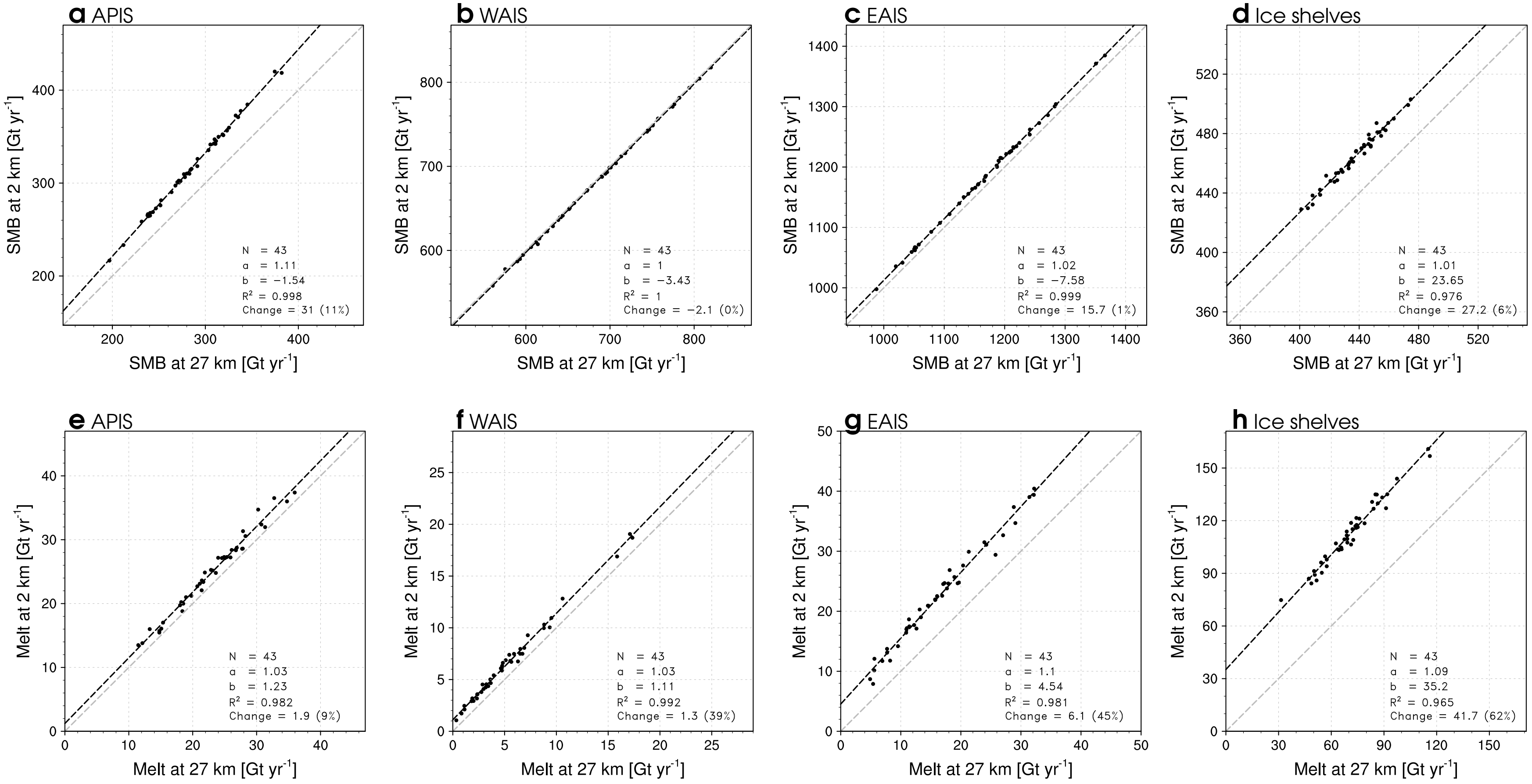

Supplementary Figure 3: Cross-model correlation between SMB and surface melt at both resolutions. Cross model correlation between surface mass balance (SMB) at 2 km and 27 km, spatially integrated over **a** the Antarctic Peninsula (APIS), **b** the West Antarctic ice sheet (WAIS), **c** the East Antarctic ice sheet (EAIS), and **d** floating ice shelves. Cross model correlation between melt at 2 km and 27 km, spatially integrated over **e** the APIS, **f** WAIS, **g** EAIS, and **h** floating ice shelves. Grounded ice on Antarctic islands are also included in the APIS, WAIS and EAIS sectors. Relevant statistics including number of records (N), the slope (a) and intercept (b) of the regression line (y = ax + b), coordination coefficient (R^2), mean model difference expressed as absolute (Gt yr^{-1}) and relative (%) values are listed.

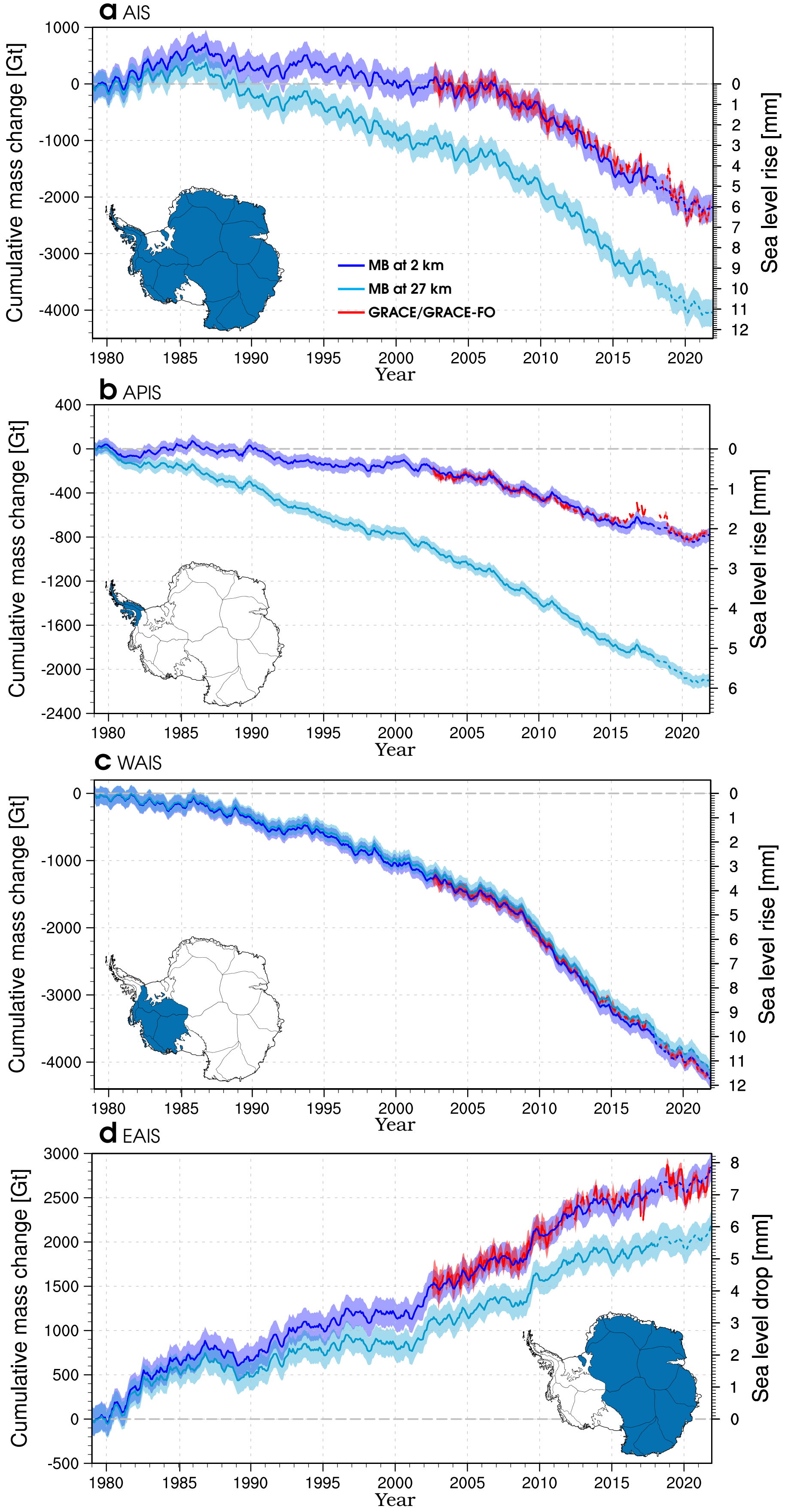

a AIS
MB at 2 km
MB at 27 km
GRACE/GRACE-FO
Cumulative mass change [Gt]
Sea level rise [mm]
Year
b APIS
c WAIS
d EAIS
Sea level drop [mm]

Supplementary Figure 4: Contemporary mass change of the grounded Antarctic ice sheet. Time series of monthly cumulative mass change (MB) at 2 km (1979-2021, blue line) and 27 km (cyan line) for **a** the grounded Antarctic ice sheet (AIS), **b** the Antarctic Peninsula (APIS), **c** the West Antarctic ice sheet (WAIS), and **d** the East Antarctic ice sheet (EAIS) sectors. Monthly mass change is estimated as surface mass balance (SMB) minus (regional) solid ice discharge from Rignot et al. (2019)[5]. As the solid ice discharge data set does not extend beyond 2017, it is linearly extrapolated thereafter. The resulting MB at both resolutions is shown as dashed lines after 2017. Regional mass change of grounded ice on Antarctic islands, i.e., detached from the grounded AIS, is included (see Methods). Mass change is converted into global sea level change assuming that 362 Gt of ice raises sea level by 1 mm. Inset maps show in blue the region of interest. Monthly mass change from GRACE/GRACE-FO is shown as red lines. Uncertainty estimates (coloured bands) are described in the Methods.

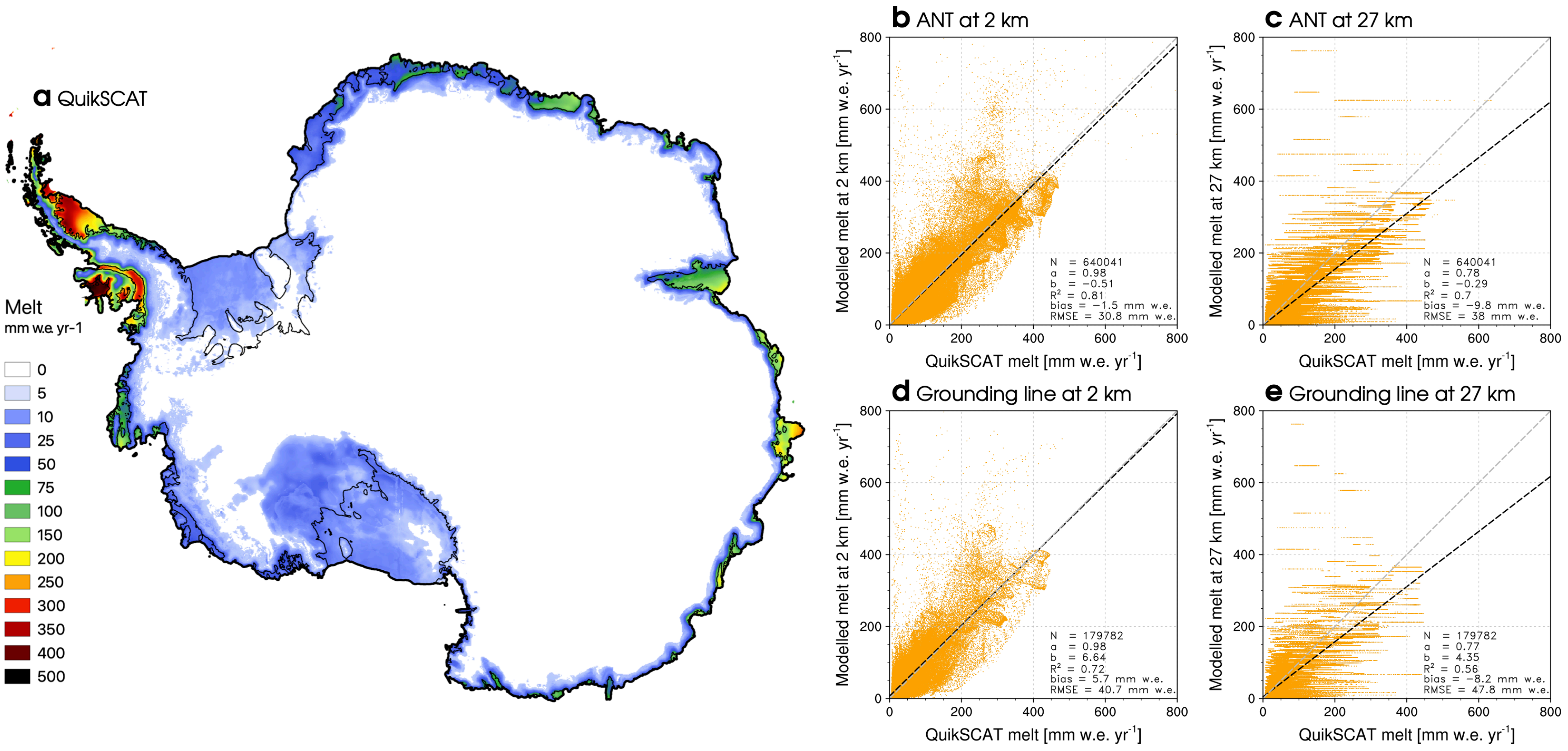

Supplementary Figure 5: Model evaluation of surface melt using remote sensing. **a** Mean annual melt from QuikSCAT at 4.45 km resolution for the period 2000-2009. Evaluation of modelled Antarctic-wide (ANT) surface melt **b** at 2 km and **c** at 27 km against QuikSCAT point records averaged for the period 2000-2009 (see Methods). **d** and **e** similar to **b** and **c** but at the vicinity of the grounding line (see Methods). Relevant statistics including number of records (N), the slope (a) and intercept (b) of the regression line (y = ax + b), coordination coefficient (R^2), mean model bias and root mean square error (RMSE) are listed.

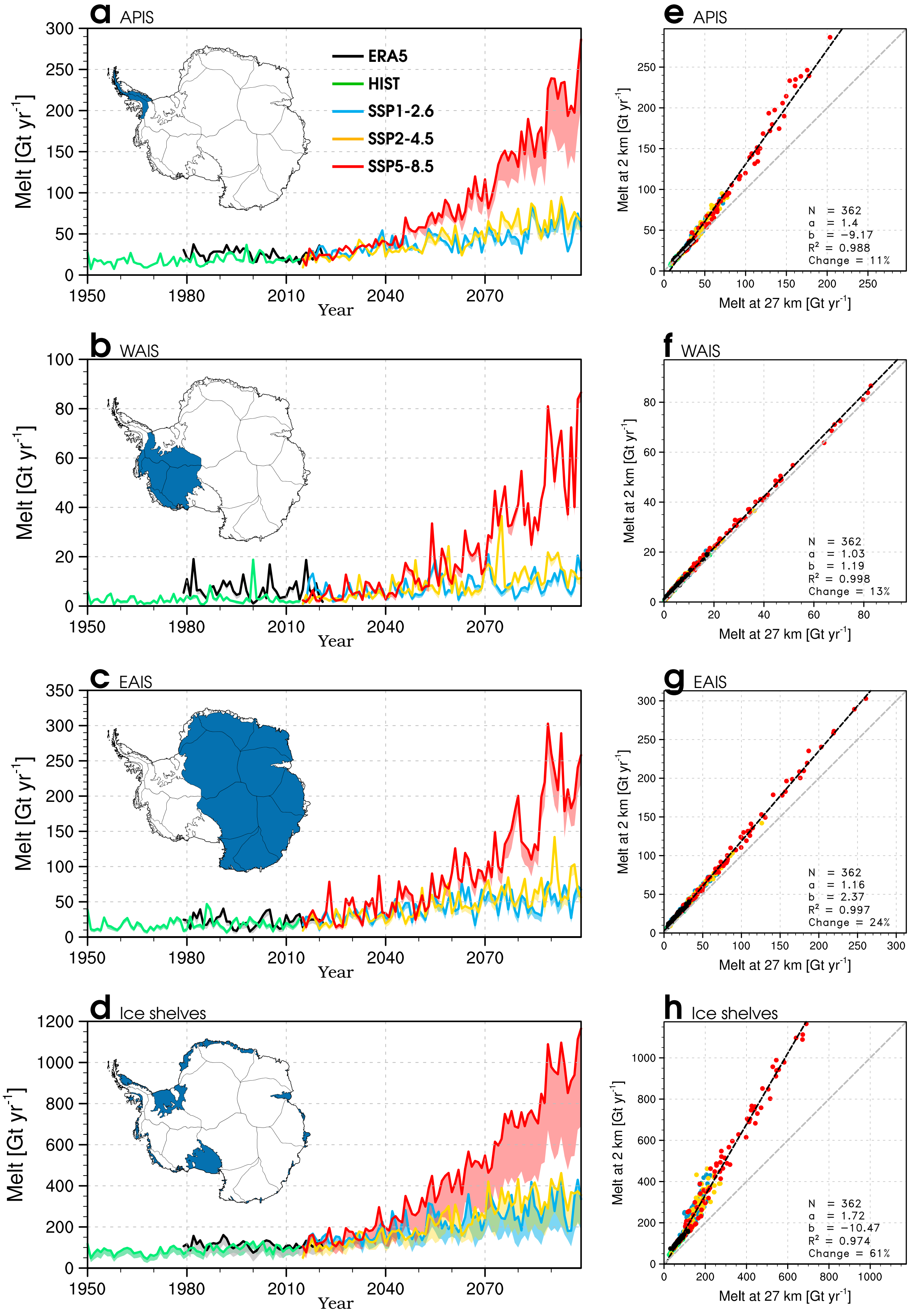
a APIS
ERA5
HIST
SSP1-2.6
SSP2-4.5
SSP5-8.5
Melt [Gt yr-1]
Year
b WAIS
c EAIS
d Ice shelves
e APIS
N = 362
a = 1.4
b = −9.17
R² = 0.988
Change = 11%
Melt at 2 km [Gt yr-1]
Melt at 27 km [Gt yr-1]
f WAIS
N = 362
a = 1.03
b = 1.19
R² = 0.998
Change = 13%
g EAIS
N = 362
a = 1.16
b = 2.37
R² = 0.997
Change = 24%
h Ice shelves
N = 362
a = 1.72
b = −10.47
R² = 0.974
Change = 61%

Supplementary Figure 6: Regional surface melt increase at both resolutions by 2100. Time series of annual surface melt integrated over **a** the Antarctic Peninsula (APIS), **b** the West Antarctic ice sheet (WAIS), **c** the East Antarctic ice sheet (EAIS) sectors and **d** floating ice shelves for the period 1950-2099. Grounded ice on Antarctic islands are also included in the APIS, WAIS and EAIS sectors. Coloured lines represent surface melt statistically downscaled to 2 km from RACMO2.3p2 forced by ERA5 reanalysis (black, 1979-2021), and by CESM2 for the historical period (green, 1950-2014), and three scenario projections under SSP1-2.6 (cyan), SSP2-4.5 (yellow) and SSP5-8.5 (red, 2015-2099). Coloured bands represent the difference with the corresponding simulations from RACMO2.3p2 at 27 km resolution. Cross model correlation of surface melt integrated over each sector between the 2 km and 27 km products is shown in **e-h**. Relevant statistics including number of records (N), the slope (a) and intercept (b) of the regression line (y = ax + b), coordination coefficient (R^2), and mean model difference expressed as a relative (%) value are listed.

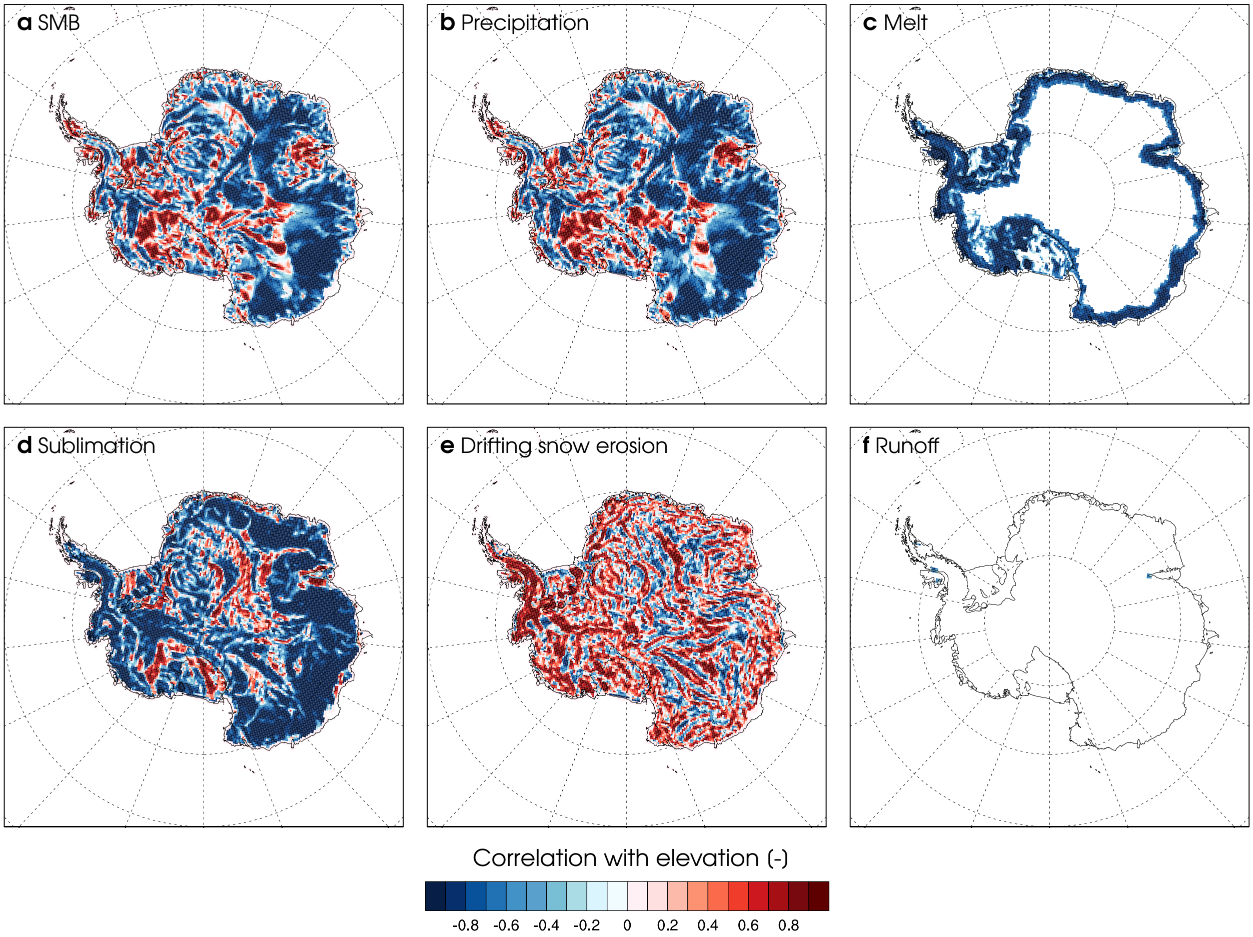

Supplementary Figure 7: Spatial correlation with elevation. Spatial correlation between annual modelled surface mass balance (SMB) and its components averaged for 1979-2021 and surface elevation prescribed in RACMO2.3p2 at 27 km. SMB and components include **a** SMB, **b** total precipitation, **c** surface melt, **d** total sublimation, **e** drifting snow erosion and **f** runoff. Spatial correlation is estimated using annual cumulative values over the current and its eight adjacent grid-cells. A positive/negative correlation means that the SMB component increases/decreases with elevation. Note that in the present-day climate, runoff is spatially limited with high local negative correlations on Larsen B and C, Amery, and Wilkins ice shelves.

Supplementary Table 1: Contemporary regional surface mass balance estimates. Annual mean integrated surface mass balance (SMB) derived from the downscaled product at 2 km and the native RACMO2.3p2 product at 27 km (1979-2021). Estimates are provided for each individual sectors, including the Antarctic Peninsula (APIS), the West Antarctic ice sheet (WAIS), the East Antarctic ice sheet (EAIS), the grounded Antarctic ice sheet (AIS) and floating ice shelves, and Antarctic-wide (ANT). For individual sectors and the AIS, Antarctic islands are included. Uncertainties are estimated as one standard deviation around the mean for the period 1979-2021. Differences between the 2 km and 27 km product are expressed as absolute (Gt yr^{-1}) and relative (%) values.

1979-21	Units	APIS	WAIS	EAIS	AIS	Ice Shelves	ANT
2 km	Gt yr^{-1}	316 $\pm$ 46	683 $\pm$ 68	1181 $\pm$ 90	2180 $\pm$ 107	465 $\pm$ 18	2645 $\pm$ 120
27 km	Gt yr^{-1}	285 $\pm$ 41	685 $\pm$ 68	1165 $\pm$ 88	2135 $\pm$ 104	438 $\pm$ 18	2573 $\pm$ 117
Δ (%)	Gt yr^{-1}	+31 (11%)	-2 (0%)	+16 (1%)	+45 (2%)	+27 (6%)	+72 (3%)

Supplementary Table 2: Regional contribution to global sea-level rise since 2002. Cumulative regional contribution to global sea-level rise expressed in mm sea-level equivalent (SLE) for the period 2002-2021. Estimates are provided for each individual sectors, i.e., the Antarctic Peninsula (APIS), the West Antarctic ice sheet (WAIS), the East Antarctic ice sheet (EAIS), and the grounded Antarctic ice sheet (AIS), including Antarctic islands. Uncertainties are estimated based on combined regional surface mass balance (SMB) and solid ice discharge (D) uncertainties (see Methods). Differences between the 2 km and 27 km product are expressed as absolute (mm SLE) and relative (%) values. It is assumed that 362 Gt of ice raises global sea level by 1 mm.

2002-21	Units	APIS	WAIS	EAIS	AIS
2 km	mm SLE	1.8 $\pm$ 0.2	8.1 $\pm$ 0.4	-3.6 $\pm$ 0.4	6.3 $\pm$ 0.7
27 km	mm SLE	3.5 $\pm$ 0.2	7.9 $\pm$ 0.4	-2.8 $\pm$ 0.4	8.6 $\pm$ 0.7
Δ (%)	mm SLE	-1.7 (49%)	+0.2 (3%)	-0.8 (29%)	-2.3 (27%)

Supplementary Table 3: Contemporary regional surface melt estimates. Annual mean integrated surface melt derived from the downscaled product at 2 km and the native RACMO2.3p2 product at 27 km (1979-2021). Estimates are provided for each individual sectors, including the Antarctic Peninsula (APIS), the West Antarctic ice sheet (WAIS), the East Antarctic ice sheet (EAIS), the grounded Antarctic ice sheet (AIS) and floating ice shelves, and Antarctic-wide (ANT). For individual sectors and the AIS, Antarctic islands are included. Uncertainties are estimated as one standard deviation around the mean for the period 1979-2021. Differences between the 2 km and 27 km product are expressed as absolute (Gt yr^{-1}) and relative (%) values.

1979-21	Units	APIS	WAIS	EAIS	AIS	Ice Shelves	ANT
2 km	Gt yr^{-1}	25 ± 6	7 ± 4	22 ± 8	54 ± 11	113 ± 19	167 ± 31
27 km	Gt yr^{-1}	23 ± 6	6 ± 4	16 ± 8	45 ± 10	71 ± 17	116 ± 29
Δ (%)	Gt yr^{-1}	+2 (9%)	+1 (39%)	+6 (45%)	+9 (36%)	+42 (61%)	+51 (46%)

Supplementary References

1. Howat, I., Porter, C., Smith, B. E., Noh, M.-J. & Morin, P. The Reference Elevation Model of Antarctica. *The Cryosphere* **13**, 665 – 674 (2019).

2. van Wessem, J. *et al.* Modelling the climate and surface mass balance of polar ice sheets using RACMO2 – Part 2: Antarctica (1979–2016). *The Cryosphere* **12**, 1479 – 1498 (2018).

3. Wang, Y. *et al.* The AntSMB dataset: a comprehensive compilation of surface mass balance field observations over the Antarctic Ice Sheet. *Earth Syst. Sci. Data* **13**, 3057 – 3074 (2021).

4. Jakobs, C. *et al.* A benchmark dataset of in situ Antarctic surface melt rates and energy balance. *Journal of Glaciology* **66**, 291 – 302 (2020).

5. Rignot, E. *et al.* Four decades of Antarctic Ice Sheet mass balance from 1979-2017. *PNAS* **116**, 1095 – 1103 (2019).
